# TRACE: A Framework for Integrating Transcript Relevance Into ACMG/AMP Variant Interpretation

**DOI:** 10.1155/humu/1095011

**Published:** 2026-09-06

**Authors:** Himanshu Goel

**Affiliations:** ^1^ Hunter Genetics, Hunter New England Local Health District, Waratah, New South Wales, Australia; ^2^ School of Medicine and Public Health, University of Newcastle, Callaghan, New South Wales, Australia, newcastle.edu.au

**Keywords:** ACMG/AMP, alternative splicing, MANE, pext, PSI, PVS1, rare disease, transcript, variant interpretation, variants of uncertain significance

## Abstract

**Background:**

Accurate clinical variant interpretation depends on the transcript used for annotation and consequence assessment. Transcript‐aware reasoning is also incorporated into existing ClinGen guidance for loss‐of‐function, splicing, functional and computational evidence and into gene‐ and disease‐specific specification. However, limited guidance exists when unresolved transcript context warrants escalation beyond routine annotation, how heterogeneous transcript‐relevance evidence should be integrated or how the resulting determination should be documented.

**Methods:**

We propose TRACE (Transcript Relevance Assessment for Clinical Evaluation), a four‐tiered gated framework. Applicable gene‐ or disease‐specific Variant Curation Expert Panel (VCEP) specifications take precedence where they resolve the transcript question. Otherwise, TRACE begins with standard transcript annotation, escalates to focused transcript‐relevance assessment only when transcript context could materially alter molecular consequence or criterion applicability and reserves targeted developmental, RNA, protein or functional evidence for unresolved cases.

**Results:**

TRACE separates transcript‐context assessment from criterion‐specific evidence assignment. Transcript context may establish whether the biological prerequisite for PVS1, PM1, PM4, PS3/BS3 or PP3/BP4 assessment is satisfied; established ClinGen SVI or gene‐specific guidance then determines whether the criterion is applied and at what strength. Representative mechanisms include exon utilisation in *TTN*, poison‐exon regulation in *SCN1A*, promoter‐specific isoforms in *DMD* and regulatory transcript architecture in *FKRP*. A *YAP1* example demonstrates withholding PVS1 when an alternative transcript preserves a downstream product, whereas *CDKL5* illustrates a historical diagnosis missed through transcript selection and now governed by gene‐specific expert curation.

**Conclusions:**

TRACE is an escalation and documentation framework, not a parallel evidence‐weighting system. Its primary aim is to make clinically material transcript determinations explicit, reproducible and auditable while preserving established SVI/VCEP rules for evidence application and strength. Whether TRACE improves classification accuracy, interlaboratory concordance or diagnostic yield requires empirical validation.

## 1. Introduction

Accurate interpretation of genomic variation is central to rare disease diagnosis. Since publication of the ACMG/AMP variant classification framework in 2015, clinical laboratories have adopted increasingly standardised approaches for assessing pathogenicity, improving consistency across institutions and enabling more reproducible genomic reporting [1]. Despite these advances, one fundamental aspect of variant interpretation often remains implicit: the transcript used for annotation and consequence prediction [2, 3]. Transcript selection is an underappreciated source of variability in variant interpretation. Most human genes generate multiple transcript isoforms through alternative splicing, alternative promoter usage and alternative polyadenylation. These isoforms can differ substantially in exon composition, protein structure, tissue distribution, developmental timing and biological function [4, 5]. Consequently, the functional impact of a variant depends not only on its genomic location but also on the transcript through which it is interpreted. A variant predicted to cause loss of function in one transcript may affect a lowly expressed exon or a biologically irrelevant isoform, whereas pathogenic variants affecting disease‐critical transcripts may be underestimated when interpretation is restricted to a single reference transcript.

The Matched Annotation from NCBI and EMBL‐EBI (MANE) project has substantially improved transcript standardisation by providing harmonised reference transcripts for most protein‐coding genes [6]. However, standardisation does not necessarily identify the transcript most relevant to disease biology. Increasing evidence demonstrates that pathogenic mechanisms may depend on tissue‐specific exon utilisation, developmental isoform switching, poison exon regulation, promoter‐specific transcription or complex non‐coding transcript architecture. In such settings, reliance on a single reference transcript may incompletely capture the biological consequences of genetic variation.

Transcript‐aware reasoning is already embedded in current variant‐interpretation guidance. The ClinGen PVS1 recommendations require consideration of nonsense‐mediated decay (NMD), whether the affected exon is present in a biologically relevant transcript or region, and specific terminal‐exon and rescue scenarios [2]. ClinGen recommendations also address predicted and observed splicing effects [3], functional evidence [7] and calibrated computational evidence [8]. Gene‐ and disease‐specific VCEP specifications may add further transcript or exon rules. TRACE therefore does not claim transcript awareness itself as novel.

At the same time, transcriptomic resources have expanded rapidly. ClinGen transcript curation identifies clinically relevant transcripts for disease‐associated genes [6, 9, 10], whereas the Genotype‐Tissue Expression (GTEx) project [11], the proportion expressed across transcripts (pext) [12] and BrainSpan developmental data [13] have substantially improved our ability to identify biologically relevant transcripts. Long‐read sequencing has further revealed a level of transcript diversity that was previously unrecognised, identifying numerous novel isoforms across disease‐associated genes [12, 14]. These resources make increasingly detailed transcript assessment possible but do not remove the need for disease‐ and mechanism‐specific judgement. The remaining implementation gap is narrower: Existing guidance and resources are distributed across criteria, genes and data types, and no single cross‐criterion workflow defines when routine transcript annotation is sufficient, when a material transcript ambiguity warrants escalation, how conflicting transcript‐context evidence should be handled or how the resulting determination should be documented. The objective is therefore not to create a new source of pathogenic evidence, but to make the antecedent transcript‐context decision more explicit and reproducible.

**Figure 1:**
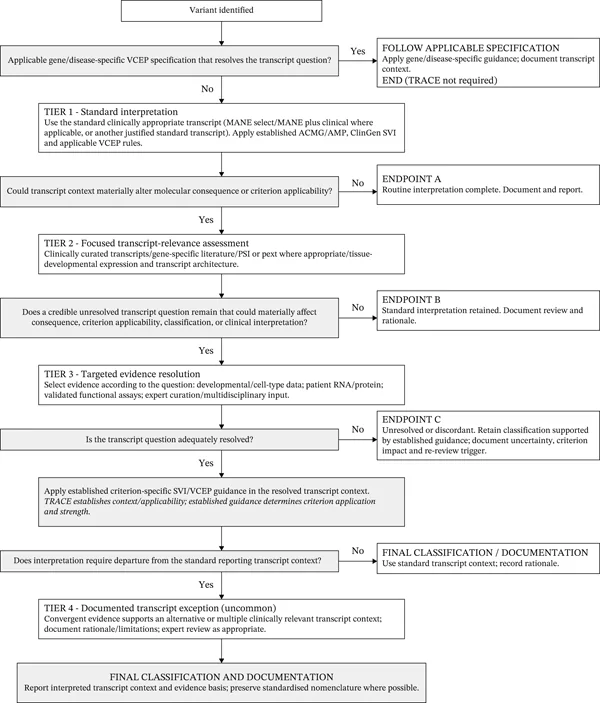
The TRACE framework workflow. Variants are annotated and classified on MANE Select (Tier 1), at which most are resolved. When the phenotype is discordant with the canonical transcript, tissue‐specific expression and pext (Tier 2), then integrated developmental, functional, single‐cell and curation evidence (Tier 3) identify the disease‐relevant transcript. A documented override of MANE Select is permitted only under multidisciplinary review when independent evidence converges (Tier 4). The guiding principle, ‘MANE First, Biology Second’, retains MANE Select as the default reporting transcript throughout. Negative determinations at Tiers 1 and 2 terminate in a single documented endpoint: classify on MANE Select, record that transcript review was considered, and stop. This schematic was prepared with the assistance of a generative artificial intelligence tool and was verified by the author; it presents no experimental data.

To address this challenge, we propose TRACE (Transcript Relevance Assessment for Clinical Evaluation), a gated four‐tiered framework for recognising and resolving transcript ambiguity. TRACE begins with standardised transcript annotation and applicable gene‐ or criterion‐specific guidance, escalates only when transcript context could plausibly alter molecular consequence or criterion applicability and provides explicit stopping rules when further assessment is unlikely to change interpretation (Figure 1). TRACE is subordinate to existing ClinGen SVI and gene/disease‐specific VCEP specifications. Where those specifications resolve the transcript question, it govern. Where uncertainty remains, TRACE provides a structured procedure for deciding whether further transcript assessment is justified, evaluating the relevant biological context, handling discordant evidence and documenting the conclusion. TRACE therefore separates transcript‐context assessment from evidence‐strength assignment: It establishes context and criterion eligibility, whereas existing criterion‐specific guidance determines whether a criterion is applied and at what strength.

In this review, we examine the biological basis and major resources for transcript‐aware interpretation, define the TRACE workflow, illustrate its relationship to selected ACMG/AMP criteria and disease mechanisms and outline practical implementation and validation strategies.

## 2. Biological Basis of Transcriptomic Functional Diversity

### 2.1. Alternative Splicing as a Major Source of Functional Diversity

Alternative splicing is a major mechanism of gene regulation, affecting more than 95% of multiexonic human genes and greatly expanding transcriptomic and proteomic diversity [4, 5]. Through exon inclusion or exclusion, alternative splice‐site selection, intron retention and mutually exclusive exon usage, individual genes can produce transcripts with distinct structural and functional properties.

The consequences of alternative splicing extend beyond protein diversity. Isoforms may differ in transcript stability, susceptibility to NMD, translational efficiency, subcellular localisation and protein interactions. Consequently, the molecular effect of a variant may depend on the transcript in which it occurs, challenging the assumption that disruption of one transcript is equivalent to disruption of all gene products.

### 2.2. Tissue and Cell Type–Specific Transcript Expression

Transcript expression is highly context dependent. Alternative promoters, differential splicing programmes and regulatory networks generate transcript repertoires tailored to individual tissues and cell types [11]. As a result, transcripts abundant in one tissue may be absent or minimally expressed in another.

This has direct implications for disease interpretation. Variants affecting highly expressed transcripts within disease‐relevant tissues are more likely to have functional consequences than variants affecting transcripts with limited biological utilisation. The importance of transcript context is particularly evident in disorders of the brain, retina, heart and skeletal muscle, where expression patterns differ substantially between tissues and developmental stages.

Single‐cell transcriptomic studies have further demonstrated that transcript usage may vary among cellular populations within the same tissue, providing an additional layer of biological complexity relevant to disease mechanisms and phenotypic heterogeneity [15].

### 2.3. Developmental Isoform Switching

Transcript expression is dynamic throughout development. Many genes undergo developmental isoform switching, whereby distinct transcripts predominate during fetal and postnatal stages [13]. Such transitions are especially prominent within the developing nervous system, where temporal regulation of transcript usage contributes to neuronal differentiation, synaptic maturation and circuit formation.

Developmental regulation has important clinical consequences because disease‐associated transcripts may be expressed only during critical developmental windows. Variants affecting fetal‐enriched transcripts can therefore contribute to congenital or neurodevelopmental disorders despite limited expression in mature tissues, highlighting a key limitation of relying solely on adult transcriptomic datasets.

### 2.4. Transcript Architecture Beyond Coding Exons

Clinically relevant transcript biology extends beyond protein‐coding exons. Alternative promoters, untranslated regions (UTRs), poison exons, deep intronic pseudoexons and other regulatory elements influence transcript abundance, processing and function. Poison exons, for example, regulate gene expression through NMD, whereas promoter‐specific transcripts may exhibit distinct tissue distributions despite sharing coding sequence.

Long‐read sequencing has revealed that transcript complexity is substantially greater than previously appreciated, identifying numerous previously unannotated isoforms and transcript structures across disease‐associated genes [12, 14]. These findings have expanded the spectrum of pathogenic mechanisms beyond conventional coding‐sequence disruption to include transcript‐specific regulatory dysfunction.

### 2.5. Implications for Clinical Genomics

Alternative splicing, tissue‐specific expression, developmental regulation and complex transcript architecture collectively determine how genomic variation is translated into molecular and clinical phenotypes. Transcript diversity therefore represents a fundamental layer of biological regulation rather than a secondary annotation feature.

As transcript‐dependent disease mechanisms become increasingly recognised, clinical genomics faces a practical challenge: How should transcriptomic evidence be incorporated into variant interpretation? Addressing this question requires resources capable of characterising transcript expression, exon utilisation and biological relevance across tissues, developmental stages and cellular contexts. The following section reviews the major transcriptomic resources that now make transcript‐aware interpretation feasible.

## 3. Transcriptomic Resources for Clinical Variant Interpretation

### 3.1. From Transcript Annotation to Transcript Relevance

The rapid expansion of transcriptomic datasets has transformed our ability to evaluate transcript relevance in human disease. Historically, transcript selection relied largely on reference annotations and expert knowledge. Today, multiple complementary resources provide empirical evidence regarding transcript expression, exon utilisation, developmental regulation and biological function, enabling transcript‐aware interpretation that moves beyond transcript existence toward assessment of transcript relevance. No single resource is sufficient in isolation; disease‐relevant transcript identification typically requires integration of expression data, transcript curation, developmental context and functional evidence. Understanding the strengths and limitations of the available resources is therefore essential for systematic incorporation of transcript biology into clinical variant interpretation.

### 3.2. MANE and Transcript Standardisation

The MANE project represents the current standard for transcript annotation in clinical genomics [6]. MANE Select provides a harmonised transcript for most protein‐coding genes, ensuring consistent nomenclature across major reference databases and reducing interlaboratory discrepancies in variant reporting.

The importance of MANE lies in standardisation rather than biological prioritisation. Although MANE Select frequently corresponds to a clinically relevant transcript, it is not designed to identify the disease‐associated isoform in every biological context. Recognition of this limitation has led to the development of MANE Plus Clinical transcripts, which capture clinically important exons or isoforms not adequately represented by the Select transcript [6]. Consequently, MANE provides the essential starting point for variant interpretation but should not be viewed as definitive evidence of transcript relevance.

### 3.3. ClinGen Transcript Curation

ClinGen transcript curation provides a structured approach to identifying transcripts that are clinically relevant for specific disease genes [9, 10]. Through expert review of transcript structure, expression patterns, functional evidence and disease‐associated variants, ClinGen panels classify exons and transcripts according to their clinical significance.

Unlike reference‐transcript annotation, transcript curation explicitly addresses biological and clinical relevance. Where expert transcript curation or a gene‐specific specification already resolves the relevant transcript question, TRACE requires no independent reassessment. Its role is confined to situations in which transcript relevance remains uncertain or must be established from multiple biological evidence resources.

### 3.4. GTEx and Exon Utilisation Metrics: Percent Spliced in (PSI) and Pext

The GTEx project has generated comprehensive transcriptomic profiles across a wide range of human tissues, providing an unprecedented resource for evaluating tissue‐specific expression [11]. Building on GTEx, the pext metric estimates the relative contribution of individual exons to total gene expression within specific tissues [12]. Pext is useful for flagging regions with little evidence of expression, but it is a derived metric and should not be treated as a universal pathogenicity threshold, particularly for developmental or cell type–restricted disorders.

PSI and pext are related but distinct. PSI estimates the proportion of transcripts that include a given exon, commonly from splice‐junction reads, whereas pext is an expression‐weighted consequence annotation derived from isoform‐level quantification [12]. PSI is the metric used in cardiac variant curation, where truncating variants affecting exons with a cardiac PSI below approximately 90% are not treated as conferring loss of function. In *TTN*, cardiac exon inclusion measured by PSI is a well‐established interpretive variable: DCM‐associated truncating variants are enriched in highly included exons, and a PSI greater than 0.9 is commonly used to denote high‐use cardiac exons [16]. Neither PSI nor pext should be applied as a universal threshold outside the context in which it has been validated.

### 3.5. Developmental Transcriptomic Resources

Many disease‐associated transcripts exhibit developmental regulation that is not adequately captured by adult tissue expression datasets. Resources such as BrainSpan provide transcriptomic data across multiple stages of human brain development and have become increasingly valuable for interpreting neurodevelopmental disorders [13]. Developmental transcriptomic data are especially important when evaluating congenital and paediatric disorders, where pathogenic transcripts may be expressed predominantly during fetal development; reliance solely on adult expression resources may therefore underestimate the relevance of developmentally restricted isoforms. As developmental transcriptomic datasets continue to expand, they increasingly provide a critical layer of evidence for transcript prioritisation in rare disease diagnostics.

### 3.6. Computational Approaches to Transcript Prioritisation

Computational methods are increasingly used to integrate transcriptomic and genomic information for variant interpretation. Tools such as SpliceAI predict the impact of variants on splicing [17], whereas CADD [18–20] and AlphaMissense [21, 22] provide complementary assessments of variant deleteriousness. Although these tools were not developed specifically for transcript prioritisation, their outputs can provide important supporting evidence when interpreted within an appropriate transcript context. Emerging machine learning approaches are increasingly integrating expression data, transcript structure, developmental information and phenotype similarity to identify disease‐relevant transcripts and prioritise candidate variants. These developments suggest that transcript‐aware interpretation may become progressively more automated, although expert review remains essential for clinical decision‐making.

### 3.7. From Resources to Interpretation

Together, these resources provide complementary perspectives on transcript relevance. MANE establishes a standardised reference transcript, ClinGen identifies clinically relevant isoforms, GTEx and pext quantify tissue utilisation, developmental and single‐cell datasets provide biological context, and computational approaches offer scalable methods for evidence integration. The challenge is no longer the absence of transcriptomic information but the lack of a structured framework for incorporating these diverse data types into clinical variant classification. The following section introduces TRACE, a hierarchical framework designed to address this implementation gap while preserving the consistency afforded by MANE‐based annotation. Table 1 shows the TRACE framework at a glance.

**Table 1:** The TRACE framework at a glance.

Tier	Question addressed	Evidence sources	Output	Expected frequency
Tier 1: Standard interpretation	Which transcript is used for annotation and classification?	MANE Select; MANE Plus Clinical where designated	Variant annotated and classified on MANE Select using standard ACMG/AMP criteria.	All variants.
Tier 2: Relevance assessment	Is there reason to think the MANE transcript does not represent the disease‐relevant gene product?	MANE Plus Clinical status; exon utilisation (PSI, pext) in the disease‐relevant tissue; expert panel transcript designation	A documented determination that transcript review is or is not required; candidate transcripts identified.	Minority of variants; minutes per case.
Tier 3: Evidence integration	Does convergent independent evidence identify a different disease‐relevant transcript?	Patient‐derived RNA or protein; expert panel curation; exon utilisation; bulk tissue and developmental expression; single‐cell data; functional studies	A ranked transcript assessment with the discordance hierarchy applied.	Uncommon; escalation criteria in Section 4.7 must be met.
Tier 4: Documented transcript exception	Should interpretation proceed outside the standard reporting transcript context?	Multidisciplinary review of the Tier 3 assessment	Documented exception recording the transcripts used, the supporting and conflicting evidence, the molecular consequence in that context, and the criteria affected.	Rare.

*Note:* Tiers are cumulative. A negative determination at any tier terminates the assessment at a documented endpoint (Figure 1). Where a ClinGen Variant Curation Expert Panel specification exists for the gene, that specification governs at every tier (Section 4.3).

## 4. The TRACE Framework

### 4.1. Rationale and Design Principles

Current transcriptomic resources provide increasingly detailed information about transcript expression, exon utilisation, developmental regulation and molecular consequence. TRACE is designed to organise how this information is escalated and documented when it is material to interpretation. It does not create new ACMG/AMP criteria, independently reweight existing criteria or supersede ClinGen SVI or gene‐specific specifications.

### 4.2. Relationship to Existing ClinGen Recommendations

Several elements of transcript‐aware interpretation are already codified. PVS1 explicitly considers biologically relevant transcripts/exons, NMD and rescue scenarios [2]. ClinGen SVI provides recommendations for functional evidence [7], computational evidence [8] and predicted/observed splicing effects [3].

These frameworks should be used directly for criterion‐specific assessment. TRACE addresses the cross‐cutting workflow question that may precede them: When is transcript context sufficiently uncertain and clinically material to warrant additional assessment, and how should that determination be documented?

This distinction is most important for PVS1. TRACE does not alter the PVS1 decision tree. Instead, where the disease‐relevant transcript or exon is not already established, TRACE provides a structured way to determine and document that input. Once the relevant context is established, the existing PVS1 framework determines whether PVS1 applies and, if so, at what strength. The same principle applies to other criteria discussed below.

For some criteria TRACE contributes very little. For PP3/BP4, for example, the calibrated thresholds remain those of existing guidance; TRACE adds only the requirement to identify and record the transcript consequence being scored. Table 2 therefore distinguishes explicitly between what existing guidance already specifies and the narrower procedural contribution claimed for TRACE.

**Table 2:** What existing ClinGen recommendations specify, what remains unspecified and what TRACE contributes.

Criterion	Already specified by existing guidance	Question left open	TRACE contribution
PVS1	Nonsense‐mediated decay prediction; whether the exon lies in a biologically relevant or constitutively expressed region; terminal exon and pseudoexon rules; nonapplication where the exon is absent from a biologically relevant transcript [2]	How the biologically relevant transcript is determined, what evidence establishes relevance, how much is sufficient and how the determination is recorded	A defined procedure for making, evidencing and documenting the determination that the flowchart requires as an input (Tiers 1–3).
PM4	Application to in‐frame length changes; routing of splicing evidence producing length change rather than loss of function [3]	Whether the affected region is represented in the transcript that matters for the disease	The same antecedent determination, applied to the region rather than the exon.
PM1	Domain and hotspot definitions from curated sources [1]	Whether the domain is expressed in the disease‐relevant transcript, and in the relevant tissue and developmental window	An explicit requirement that domain relevance be established in the interpreted transcript before PM1 is applied.
PS3 and BS3	Assay validation, controls and calibration of evidence strength; the requirement that an assay model the disease mechanism [7]	Which isoform the assay construct should represent, and what follows when it represents another	Nonapplication where the construct represents an isoform other than the disease‐relevant transcript, rather than application at reduced strength.
PP3 and BP4	Calibrated score thresholds and the evidence strengths they support [8]	Which transcript the prediction should be generated against	A documentation requirement only. The calibrations are transcript‐agnostic in construction and TRACE does not modify them.
PS1 and PM5	Application by amino acid consequence and by established pathogenic change at the same residue [1]	Whether the compared variants were described on the same transcript	A requirement that the transcript be stated when asserting equivalence of consequence between variants.

*Note:* The contribution claimed for TRACE is confined to the antecedent transcript determination and its documentation. Where existing guidance already resolves a question, the table records that rather than restating it as a gap.

### 4.3. Relationship to Expert Panel Specifications

Where an applicable ClinGen VCEP specification addresses the transcript or exon question for a gene/disease pair, that specification takes precedence. TRACE should not be used to override or run in parallel with such rules. If a VCEP specification exists but is silent on the particular transcript ambiguity, TRACE may be used only as a compatible escalation and documentation procedure, with the VCEP rules remaining authoritative for criterion application and strength.

Accordingly, transcript‐sensitive genes such as *TTN*, *DMD* and *CDKL5* are used in this review to illustrate biological principles or historical failure modes, not to imply that TRACE supersedes current gene‐specific guidance. The curator should verify current specifications at the time of interpretation because expert‐panel guidance evolves.

### 4.4. Overview of the Four‐Tier Framework

TRACE is a gated four‐tier workflow (Figure 1 and Table 1), not a requirement that every variant undergo progressively more complex transcript analysis. Before TRACE is entered, applicable gene‐ or disease‐specific specifications are checked. Tier 1 performs standard interpretation in the appropriate reporting transcript context. Tier 2 is triggered only when transcript choice could materially alter molecular consequence or criterion applicability. Tier 3 uses targeted additional evidence to resolve a credible remaining ambiguity. Tier 4 is an uncommon, documented transcript exception when convergent evidence justifies interpretation outside the standard reporting transcript context. Every branch has an explicit stopping point.

### 4.5. Tier 1: Standard Interpretation

Tier 1 begins with the transcript specified by applicable gene‐ or disease‐specific guidance. Where no such specification resolves the question, MANE Select, supplemented by MANE Plus Clinical where designated, provides the preferred standardised starting point [6]. Routine ACMG/AMP and ClinGen SVI assessment is completed without further TRACE escalation when transcript context is biologically coherent and no plausible transcript‐dependent alternative would alter consequence or criterion applicability.

### 4.6. Tier 2: Identifying When Transcript Review Is Required

Tier 2 is triggered by a defined transcript‐related question rather than applied routinely. Triggers include alternative exon or promoter usage, tissue‐ or developmental stage–specific biology, poison‐exon mechanisms, discordance between the standard annotated consequence and the established disease mechanism, strong phenotype‐gene concordance despite an apparently non‐coding consequence or uncertainty about whether a functional domain or assay corresponds to the relevant isoform. Assessment is focused and may use clinically curated transcripts, gene‐specific literature, PSI or pext where appropriate and tissue/developmental expression. A positive Tier 2 screen identifies a material ambiguity; it does not itself assign an ACMG/AMP criterion.

### 4.7. Triage, Escalation and Stopping Rules

Tier 2 is designed as a focussed desk‐based assessment using resources already available to the curator. Escalation to Tier 3 is appropriate when (i) a credible transcript‐related ambiguity remains; (ii) resolving it could materially alter molecular consequence, criterion applicability, final classification, diagnostic interpretation or another clinically meaningful conclusion; and (iii) evidence of sufficient quality is reasonably available or obtainable. Immediate management change is not required for escalation, and Tier 3 need not always be completed within the initial reporting window.

Where potentially decisive evidence cannot be obtained within the initial reporting period, the current classification may be issued with the transcript uncertainty documented and a defined re‐review trigger. Conversely, escalation should stop when additional transcript assessment is unlikely to alter interpretation, suitable evidence is unavailable or uncertainty persists despite appropriate investigation.

Stopping is therefore an explicit TRACE outcome rather than a failure to complete the framework. Most variants should stop at Tier 1; Tier 2 should remain focused; Tier 3 should be uncommon and question‐driven.

### 4.8. Tier 3: Evidence Integration and Transcript Prioritisation

When transcript ambiguity remains after Tier 2. Tier 3 selects evidence according to the biological question rather than requiring a fixed panel of investigations. Relevant sources may include

• tissue‐ and developmental stage–specific expression, exon utilisation or splice‐junction evidence, including PSI or pext where appropriate
• patient‐derived RNA or protein studies from a disease‐relevant tissue or justified surrogate;
• validated functional assays that model the relevant isoform and disease mechanism;
• expert transcript curation and gene‐specific literature; and
• phenotype and mechanism concordance.

Evidence sources are not placed in a universal rank order. Their persuasiveness depends on biological proximity to the disease mechanism, tissue and developmental relevance, technical validity, directness and independence. A well‐designed patient‐derived RNA study may be more informative than adult bulk‐tissue expression for a specific splice question, but a negative assay using an irrelevant tissue or isoform should not override robust biological evidence.

### 4.9. Integration With ACMG/AMP Criteria

TRACE informs the context in which existing ACMG/AMP criteria are assessed; it does not introduce transcript‐specific evidence codes or strength modifiers (Table 3). Transcript context may show that a biological prerequisite for a criterion is absent, present or unresolved. Where it is present, the criterion‐specific SVI/VCEP framework determines whether the criterion is ultimately applied and at what strength.

**Table 3:** Transcript context and the applicability of ACMG/AMP evidence criteria. Transcript evidence assessed at Tiers 2 and 3 (tissue and exon expression, developmental, functional, single‐cell and curation data) determines whether specific evidence codes may be applied: PVS1, PM4, PM1, PS3 and BS3 and PP3 and BP4. For each criterion, the table shows the transcript finding and the resulting binary determination: apply or do not apply. Transcript context governs the applicability of existing criteria and does not alter the strength at which they are applied or create new ones. This schematic was prepared with the assistance of a generative artificial intelligence tool and was verified by the author; it presents no experimental data.

Criterion	Transcript‐context finding	TRACE action	Consequence for classification	Basis
PVS1	Exon absent from the disease‐relevant transcript or gene‐specific evidence establishes insufficient relevance (for example, a low‐use *TTN* cardiac exon).	Do not apply on this basis	Biological transcript/exon prerequisite is not established.	[2, 16]
PVS1	Affected exon is established as disease‐relevant.	Proceed to the PVS1 framework	Use the existing PVS1 decision tree to determine whether PVS1 applies and at what strength.	[2]
Observed RNA/PVS1	RNA demonstrates a transcript consequence consistent with loss of function in the relevant transcript.	Route to ClinGen splicing/PVS1 assessment	Interpret the observed consequence under ClinGen splicing guidance; an RNA assay is not PS3 solely because it is experimental.	[3]
PM4	Observed/predicted in‐frame protein‐length change occurs in the disease‐relevant transcript.	Route to PM4 assessment	Consider PM4 under existing guidance; withhold if the relevant transcript does not produce the length change.	[1, 3]
PM1	Critical domain/hotspot is absent from the disease‐relevant isoform.	Do not apply	The biological premise for domain/hotspot evidence is not met in that isoform.	[1]
PS3/BS3	Assay construct/model does not represent the disease‐relevant isoform or transcript‐dependent mechanism.	Do not use for the transcript‐dependent question	The assay does not answer the biological question being curated.	[7]
PS3/BS3	Assay adequately models the relevant isoform and mechanism.	Proceed to PS3/BS3 assessment	Assay validity and evidence strength are then determined using established functional‐evidence guidance.	[7]
PP3/BP4	Prediction is generated for the relevant transcript consequence.	Assess using the established calibrated threshold	Transcript choice is recorded; TRACE does not change the calibrated threshold or strength.	[8]

*Note:* Standing rules: Where high‐quality transcript evidence remains materially discordant, TRACE does not force a transcript determination. Document the uncertainty and use applicable ClinGen SVI or gene‐specific guidance conservatively. If that guidance requires biological transcript/exon relevance to be established and it cannot be established, withhold the criterion. Applicable VCEP specifications govern.

For PVS1, an exon or transcript shown to be irrelevant to the disease mechanism cannot support the required loss‐of‐function premise. Where biological relevance is established, the variant proceeds through the existing PVS1 decision tree, unmodified [2].

For PP3 and BP4, computational predictions should be generated for the relevant transcript consequence and interpreted using the established calibrated thresholds; transcript context does not change those thresholds [8, 17].

For PM1, the criterion is considered only when the critical functional domain or mutational hotspot is represented in the disease‐relevant isoform. Where it is not, the criterion is not applied.

For PS3/BS3, functional evidence is applied only when the experimental system represents the disease‐relevant transcript. Where the assay construct represents a different isoform, the criterion is not applied, rather than applied at reduced strength [7].

For PM4 and observed splicing consequences, the relevant transcript must actually produce the in‐frame length change or loss‐of‐function consequence under consideration. Observed RNA effects are routed according to the molecular consequence under ClinGen splicing guidance rather than treated as PS3 merely because they were experimentally measured [3].

### 4.10. Tier 4: Document Transcript Exception

Tier 4 is reserved for uncommon situations in which convergent evidence justifies interpretation outside the standard reporting transcript context, including cases with an alternative or multiple disease‐relevant transcripts. A Tier 4 decision should document the transcript(s) used, the supporting and conflicting evidence, the molecular consequence in that context and the affected ACMG/AMP criteria. Expert or multidisciplinary review is appropriate when the decision has substantial diagnostic or management implications.

A Tier 4 transcript decision is not itself pathogenic evidence and does not confer an ACMG/AMP strength. Standardised nomenclature should be preserved where possible, with the interpreted transcript context stated additionally when it differs from the reporting transcript.

### 4.11. Implementation in Diagnostic Workflows

TRACE is intended to be implementable within existing laboratory workflows rather than requiring a dedicated platform. Practical implementation could include a structured field recording the transcript used for interpretation and the reason, annotation pipelines that retain consequences on clinically relevant transcripts, a concise transcript‐rationale field and inclusion of transcript‐context decisions in laboratory standard operating procedures and audit.

When a nonstandard transcript materially affects interpretation, reports and database submissions should identify the transcript context clearly and follow the nomenclature and submission conventions of the relevant laboratory, database and gene‐specific guidance. TRACE does not prescribe a universal ClinVar submission transcript or require duplicate HGVS reporting in every case.

The minimum implementation requirement is therefore documentary: A reader should be able to determine which transcript context was used, why additional review was triggered, what evidence was considered, what remained uncertain and which criterion‐specific guidance governed the final classification.

### 4.12. Genes Without a MANE Transcript and Other Edge Cases

A framework whose standard starting transcript is unavailable or biologically inappropriate must allow a justified alternative. Where an applicable VCEP or other authoritative gene‐specific specification designates the transcript, that designation should be followed. Otherwise, a stable RefSeq or Ensembl transcript may be selected according to disease biology and laboratory policy, with the choice documented. MANE Plus Clinical should be used where designated within the MANE framework; it should not be treated as a generic fallback independent of MANE [6].

Where multiple transcripts are independently clinically relevant, consequences should be evaluated across those transcripts rather than forcing a single transcript to be declared biologically correct. For complex loci, locus‐specific guidance takes precedence. For non‐coding RNA genes, protein‐coding assumptions underlying criteria such as PVS1 or PM4 are not directly transferable and mechanism‐specific evidence is required.

Adult bulk‐tissue resources also require caution in developmental disorders: Low adult expression should not establish biological irrelevance when the mechanism operates during fetal development or in a restricted cell population.

### 4.13. Governance and Validation

Because transcript interpretation introduces additional complexity, TRACE emphasises transparency and auditability. Transcript‐dependent decisions should record the evidence supporting the selected context, material discordance and the ACMG/AMP criteria whose assessment depends on that context. Formal validation should evaluate whether this added structure improves reproducibility without imposing disproportionate curation burden [23].

### 4.14. TRACE as an Implementation Framework

TRACE is therefore an implementation and escalation framework around existing standards. Its intended contribution is to make unresolved transcript‐context decisions explicit, proportionate and auditable, while leaving criterion‐specific evidence application and strength to establish ACMG/AMP, ClinGen SVI and gene/disease‐specific guidance.

## 5. Transcript‐Aware Application of ACMG/AMP Evidence Criteria

### 5.1. Transcript Relevance as an Evidence Modifier

Several ACMG/AMP criteria depend on assumptions about transcript structure, exon inclusion, protein architecture or the experimental model used to assess function [1]. When the transcript context is wrong, the molecular premise on which a criterion depends may also be wrong. Transcript relevance should therefore be treated as context for criterion‐specific assessment rather than as a new pathogenic evidence category.

TRACE does not assign independent odds of pathogenicity or points to transcript relevance. If the relevant transcript/exon prerequisite is not established, a criterion that depends on that prerequisite is withheld. If the prerequisite is established, the variant proceeds to the applicable SVI/VCEP framework, which determines whether the criterion is applied and at what strength. This avoids unsupported reweighting and reduces the risk of double counting the same transcript evidence twice.

### 5.2. PVS1: Transcript Context and Predicted Loss‐of‐Function Variants

PVS1 is potentially applicable only when loss of function is an established disease mechanism and the predicted loss‐of‐function consequence occurs in a biologically relevant transcript or exon. A truncating consequence in an exon that is not relevant to the disease mechanism should not be accommodated by simply lowering PVS1 strength. Conversely, establishing transcript or exon relevance permits the variant to proceed through, but does not replace, the established PVS1 decision framework, which then determines whether PVS1 is applied and at what strength. This reasoning is already embedded in the PVS1 framework, which asks whether the affected exon is present in a biologically relevant transcript and directs nonapplication where that prerequisite is not met [2]. Exon utilisation in the disease‐relevant tissue is estimated from population‐scale expression resources [11, 12].

### 5.3. PP3, BP4 and Computational Evidence

Computational prediction tools evaluate the potential impact of variants using sequence conservation, protein structure, splicing prediction and machine learning approaches. Commonly used tools include CADD [18, 19], SpliceAI [17] and AlphaMissense [24]. The ClinGen Sequence Variant Interpretation Working Group has provided calibrated recommendations for applying computational evidence to the PP3 and BP4 criteria, including the evidence strengths that different prediction thresholds support [8].

### 5.4. PS3, BS3 and Functional Evidence in the Correct Transcript Context

Functional studies provide some of the strongest evidence available for variant interpretation, and the ClinGen Sequence Variant Interpretation Working Group has established a framework for applying the functional evidence criteria PS3 and BS3, including assay validation and the calibration of evidence strength [7]. However, experimental systems frequently evaluate variants within a single transcript construct that may not correspond to the disease‐relevant isoform. This limitation is particularly important for genes exhibiting extensive alternative splicing, developmental isoform switching or promoter‐specific expression, where functional assays performed using biologically irrelevant transcripts may underestimate or misrepresent disease mechanisms.

Transcript‐aware application of PS3 and BS3, therefore, requires consideration of whether the experimental system adequately models the transcript implicated in disease. An assay using an irrelevant isoform is not assigned a weaker PS3/BS3 strength; it may simply fail to answer the transcript‐dependent question. Where the model is appropriate, assay validation and evidence calibration follow established recommendations [7]. RNA assays that establish transcript‐level consequences are addressed separately under splicing/PVS1/PM4 guidance rather than treated as PS3 by default.

### 5.5. PM1 and Functional Domain Interpretation

The PM1 criterion relies on the presence of a variant within a critical functional domain or mutational hotspot. However, the relevance of a domain may depend on transcript architecture. Alternative splicing can create transcript‐specific protein structures, resulting in domains that are present in some isoforms but absent in others; likewise, the clinical significance of a mutational hotspot depends on whether the corresponding transcript is expressed within disease‐relevant tissues. Application of PM1 should therefore consider not only the location of a variant within a protein but also the biological relevance of the transcript in which that domain is expressed.

### 5.6. RNA Evidence and Emerging Functional Data

RNA sequencing can identify aberrant splicing, exon skipping, intron retention, poison‐exon activation, allele‐specific expression and transcript degradation that may not be apparent from DNA sequencing alone [12]. Rare‐disease studies have shown that RNA analysis can resolve otherwise difficult variants and increase diagnostic yield in selected cohorts [25–27].

The evidentiary route depends on what the assay demonstrates. An RNA result can establish an observed transcript consequence; if that consequence is predicted to cause loss of function, it is evaluated under the ClinGen splicing/PVS1 framework, whereas an in‐frame protein‐length change may be relevant to PM4 [3]. RNA evidence is not PS3 solely because it is experimental; it does not necessarily demonstrate the functional effect of the resulting protein.

### 5.7. Integrating Multiple Lines of Evidence

TRACE is compatible with points‐based approaches because it does not assign independent numerical weight to transcript relevance a [28]. At present, no general likelihood ratios or odds‐of‐pathogenicity estimates have been established for transcript‐level evidence as a distinct evidence category. Assigning fixed point penalties for low pext or bonuses for high transcript expression would therefore create unsupported precision and risk double counting information already incorporated within criterion‐specific guidance.

Under TRACE, transcript context instead determines whether the biological prerequisite for criterion‐specific assessment is satisfied. If a criterion is validly applied, it contributes the point value corresponding to the strength assigned by established ACMG/AMP, ClinGen SVI or gene‐specific guidance; if the required biological premise is not met, the criterion contributes no points. TRACE therefore leaves the underlying points‐based arithmetic unchanged.

Future quantitative calibration remains possible. A reference set of transcript‐sensitive variants with expert‐curated classifications could be used to estimate the independent predictive value of specific transcript metrics, stratified by disease mechanism, tissue and developmental context and predicted susceptibility to NMD. Until such calibration is available, transcript‐specific numerical reweighting should be regarded as a research question rather than a clinical interpretation rule.

### 5.8. Two Worked Examples: Transcript Context and the Applicability of PVS1

A useful worked example should demonstrate what transcript assessment changes without implying that TRACE itself supplies pathogenic evidence. Table 4 therefore separates a formal *YAP1* example, in which transcript context prevents application of PVS1, from a historical *CDKL5* vignette, in which transcript selection determined whether a clinically relevant variant reached curation at all.

**Table 4:** Transcript context and criterion applicability at each stage of the two worked examples.

Example	Stage	Transcript	Transcript finding	Effect on criterion application	Classification
*YAP1* c.178dupG, p.(Asp60GlyfsTer52); [29]	Consequence‐only reading	NM_001130145.3 (MANE Select)	Early frameshift in the first coding exon; apparent loss of function.	A loss‐of‐function criterion would ordinarily be considered.	Not issued on this basis
*YAP1*	Transcript assessment	NM_001130145.3	Experimentally supported alternative transcription and translation start downstream of the variant, initiating at approximately Met179 [30].	The biological premise for PVS1 is not secure.	Assessment continues
*YAP1*	Published interpretation	NM_001130145.3	The variant lies upstream of the alternative start; a downstream product may be preserved.	PVS1 not applied. PS2 and PM2 applied, as reported under the 2015 framework.	Likely pathogenic, as published
*CDKL5* c.2842C > T, p.(Arg948Ter); [31]	Reported analysis	Longer legacy transcripts	Position is intronic or non‐coding; variant not returned by earlier panel and exome analyses.	No criterion assessed; the variant never reached curation.	No classification issued
*CDKL5*	Reanalysis	NM_001323289.1	Position is coding in the brain‐predominant transcript; genome sequencing established the diagnosis.	Criterion assessment becomes possible.	Reported as causative
*CDKL5*	Current status	NM_001323289.2 (MANE Select)	Gene governed by the ClinGen Rett and Angelman‐like Disorders VCEP.	VCEP specification governs criterion application and strength.	Pathogenic, ClinGen expert panel

*Note:* The two examples run in opposite directions and neither supports a transcript‐derived strength modifier. In *YAP1* transcript, the context withholds PVS1 outright: The criterion is not applied, rather than applied at reduced strength, and the remaining evidence is combined without modification. In *CDKL5*, transcript selection determined whether the variant reached curation at all; the classification itself is not a TRACE output, since the gene is governed by an expert panel specification whose rules take precedence (Section 4.3). The *YAP1* interpretation is reported as published under the 2015 framework rather than recalculated under later Sequence Variant Interpretation changes to individual evidence strengths. Expert panel status and current variant classifications should be verified against the ClinGen registry at the time of use.

#### 5.8.1. *YAP1:* Transcript Context Withholds PVS1

DeYoung et al. reported NM_001130145.3(*YAP1)*:c.178dupG, p.(Asp60GlyfsTer52) in an individual with bilateral uveal coloboma and microphthalmia [29]. On the current MANE Select transcript, the variant is an early frameshift that would ordinarily prompt consideration of a loss‐of‐function criterion. However, *YAP1* has an experimentally supported alternative transcription/translation start downstream of the variant, with a shorter product predicted to initiate at approximately Met179 [30]. The authors therefore did not apply PVS1 because the alternative transcript could preserve a downstream product. Confirmed de novo occurrence (PS2) and absence from population databases (PM2, under the 2015 framework used in that report) supported a published likely pathogenic classification [29]. The example is reported as published rather than retrospectively recalculated under later SVI changes to individual evidence strengths.

#### 5.8.2. *CDKL5*: Transcript Context Determines Whether the Variant Is Assessed

Schoch et al. described NM_001323289.1(*CDKL5)*:c.2842C > T, p.(Arg948Ter), which was missed on earlier epilepsy‐panel and exome analyses because the laboratories used older *CDKL5* transcripts in which the position was intronic/non‐coding. Genome sequencing identified the variant as coding in the brain‐predominant transcript and provided the molecular diagnosis [31]. This is a clear historical example of transcript selection determining whether a variant reaches clinical curation at all.

The current situation is different and should not be recreated as an independent TRACE classification. NM_001323289.2 is now MANE Select and is the transcript used by the ClinGen Rett and Angelman‐like Disorders VCEP. The exact c.2842C > T p.(Arg948Ter) variant is currently classified Pathogenic for CDKL5 disorder by the ClinGen expert panel. Current curation therefore follows the VCEP specification, consistent with the TRACE precedence rule. Together, the two examples show opposite safeguards: *YAP1* prevents overapplication of a LoF criterion when an alternative product may be preserved, whereas *CDKL5* shows how incomplete transcript annotation can cause a pathogenic coding variant to be missed. Neither example supports a general transcript‐derived strength modifier.

### 5.9. From Evidence Assessment to Disease Mechanism

The criteria discussed above illustrate how transcript context can determine the molecular consequence and biological applicability of ACMG/AMP evidence criteria. However, transcript‐dependent effects are most clearly appreciated when examined within specific disease paradigms. Numerous rare disease genes demonstrate mechanisms in which transcript context fundamentally alters variant interpretation, pathogenicity assessment and genotype–phenotype correlation. The following section examines representative examples spanning exon utilisation, poison‐exon regulation, developmental isoform switching, promoter‐specific transcription and non‐coding transcript architecture (Table 5).

**Table 5:** Transcript‐dependent disease mechanisms in rare disease genes.

Gene	Transcript feature	Mechanism	Consequence for interpretation	Expert panel specification
*TTN*	Extensive alternative splicing; cardiac and skeletal isoforms	Exon utilisation determines whether a truncating variant confers loss of function.	Truncating variants in exons with low cardiac utilisation are not treated as loss of function.	Yes
*SCN1A*	Developmentally regulated poison exon	Increased poison exon inclusion targets transcripts for nonsense‐mediated decay.	A non‐coding variant produces a loss‐of‐function mechanism invisible to canonical annotation.	Yes
*SCN2A*	Neonatal and adult isoform switching	Developmental transition alters channel biophysics.	Adult transcriptomic data may understate the relevance of a fetal or neonatal transcript.	Yes
*CACNA1C*	Alternatively spliced exon 8A	Developmental utilisation of the alternative exon mediates the Timothy syndrome phenotype.	Variant consequence depends on which mutually exclusive exon is affected.	No
*DMD*	Multiple promoter‐specific isoforms (Dp427, Dp140, Dp71)	Promoter usage generates tissue‐restricted products.	Variants selectively disrupting brain‐expressed isoforms shift the neurodevelopmental phenotype.	Yes
*FKRP*	Non‐coding upstream exons	Disruption of regulatory transcript processing without coding change	A variant with no coding consequence is pathogenic on RNA evidence.	No
*ABCA4*, *USH2A*	Retina‐specific exons absent from reference transcripts	Tissue‐restricted transcript architecture.	Variants classified as non‐coding on canonical annotation disrupt retinal transcripts.	*ABCA4* yes; *USH2A* yes
*CDKL5*	Brain‐predominant transcript NM_001323289.2; final 170 nucleotides of the terminal exon non‐coding in the longer transcripts	Region is coding only in the brain transcript.	Under legacy annotation, a coding variant is reported as intronic and never reaches curation; see Section 5.10.2.	Yes (Rett and Angelman‐like Disorders VCEP)

*Note:* Genes with an expert panel specification are presented as demonstrations of transcript‐dependent biology. Curation of those genes follows the specification rather than TRACE (Section 4.3). Specification status should be verified against the ClinGen criteria specification registry at the time of use.

## 6. Transcript‐Dependent Disease Mechanisms: Lessons From Rare Disease Genomics

The impact of transcript selection on variant interpretation is most apparent in disorders where disease mechanisms depend on specific transcript isoforms, developmental expression patterns or transcript‐specific regulatory elements. These examples illustrate how transcriptomic context can fundamentally alter assessments of pathogenicity, genotype–phenotype correlation and functional relevance (Table 5).

### 6.1. Exon Utilisation and Loss‐of‐Function Interpretation: *TTN*

The *TTN* gene is a prototypical example of transcript‐dependent interpretation because of extensive alternative splicing in cardiac and skeletal muscle. Roberts et al. integrated sequencing with human‐heart RNA data and showed that DCM‐associated truncating variants are enriched in exons with high cardiac inclusion, measured as PSI, whereas truncating variants in controls more often affect lower use exons [16]. Cardiac PSI greater than 0.9 is commonly used to define highly included exons in contemporary *TTN* analyses. This gene‐specific metric illustrates why exon presence alone is insufficient; it should not be generalised as a universal pext or PSI threshold for other genes.

### 6.2. Poison Exons and Regulatory Transcript Biology: *SCN1A*

Not all pathogenic mechanisms arise through disruption of protein‐coding sequence. Poison exons represent a distinct regulatory mechanism whereby alternative exon inclusion introduces a premature termination codon, targeting transcripts for NMD and reducing protein expression. In *SCN1A*, variants that increase inclusion of a poison exon can reduce functional sodium channel abundance and cause severe developmental and epileptic encephalopathy despite leaving the canonical coding sequence unchanged [32]. These findings expanded the spectrum of pathogenic variation beyond conventional coding and splice‐site variants and demonstrated how transcript regulation itself can constitute a disease mechanism.

From a diagnostic perspective, poison exon disorders highlight the limitations of transcript annotation strategies focused exclusively on protein‐coding exons. Accurate interpretation requires recognition of transcript‐specific regulatory elements and understanding of their role in gene expression.

### 6.3. Developmental Isoform Switching: *SCN2A* and *CACNA1C*

Many genes undergo developmental transitions in transcript usage, resulting in isoforms that predominate during fetal development and others that become dominant postnatally. Variants affecting these developmentally regulated transcripts may produce distinct phenotypic consequences despite occurring within the same gene.

*SCN2A* provides a well‐characterised example. Developmental switching between neonatal and adult sodium channel isoforms alters channel biophysics and influences the phenotypic effects of pathogenic variants [33]. Similarly, the pathogenic *CACNA1C* variant responsible for Timothy syndrome occurs within exon 8A, an alternatively spliced exon whose developmental utilisation contributes to disease pathogenesis [34]. These examples illustrate an important limitation of adult transcriptomic datasets. Disease‐relevant transcripts may be expressed primarily during embryonic or early postnatal development and therefore appear biologically insignificant when evaluated using adult tissues alone.

### 6.4. Promoter‐Specific Isoforms and Phenotypic Heterogeneity: *DMD*

The *DMD* gene generates multiple tissue‐specific isoforms through the use of alternative promoters. These include the full‐length dystrophin isoforms (Dp427) as well as shorter transcripts such as Dp140 and Dp71, which exhibit distinct expression patterns and biological functions [35].

This complex transcript architecture contributes directly to genotype–phenotype correlations. Variants affecting all major isoforms typically result in classic Duchenne muscular dystrophy, whereas mutations selectively disrupting shorter brain‐expressed isoforms may disproportionately influence neurodevelopmental outcomes, including intellectual disability, attention‐deficit/hyperactivity disorder and autism spectrum disorder [35]. The *DMD* paradigm demonstrates how promoter‐specific transcription can generate clinically meaningful phenotypic diversity within a single disease gene and highlights the importance of considering transcript architecture when interpreting genotype–phenotype relationships.

### 6.5. Non‐Coding Transcript Architecture and Regulatory Splicing: *FKRP*

Transcript‐dependent disease mechanisms are not confined to coding exons. The *FKRP* gene contains multiple non‐coding upstream exons that contribute to transcript regulation despite not encoding protein sequence. Saylam et al. described a pathogenic intronic variant, c.−253 + 4A > G, located within the non‐coding region of *FKRP*, which disrupted normal transcript processing and resulted in limb‐girdle muscular dystrophy when present in trans with the common pathogenic variant c.826C > A (p.Leu276Ile) [36]. RNA sequencing demonstrated marked disruption of transcript splicing despite preservation of the coding sequence. This example illustrates how transcript‐specific regulatory architecture can mediate disease and emphasises the value of RNA‐based functional studies in resolving variants that would otherwise remain difficult to interpret using coding‐sequence analysis alone.

### 6.6. Deep Retinal Transcripts and Hidden Disease Mechanisms

Inherited retinal diseases provide some of the strongest evidence that clinically relevant transcripts may not be represented adequately by canonical annotations. Deep retinal transcriptomic studies have identified previously unrecognised exons and transcript isoforms that are highly expressed in photoreceptor cells but absent from conventional reference transcripts [37–39]. These discoveries have led directly to the identification of pathogenic variants in genes such as *ABCA4* and *USH2A*, explaining cases that remained unsolved despite extensive genomic investigation. In several instances, variants initially classified as non‐coding or of uncertain significance were subsequently shown to disrupt retinal‐specific transcripts and cause disease. The retinal disease experience demonstrates how incomplete transcript annotation can obscure pathogenic mechanisms and highlights the importance of tissue‐specific transcript discovery for rare disease diagnostics.

### 6.7. Common Themes Across Transcript‐Dependent Disorders

Although these examples involve diverse genes and disease mechanisms, several common principles emerge. First, transcript relevance is frequently tissue‐specific, developmental stage‐specific or cell‐type specific. Second, pathogenic mechanisms often involve transcript regulation rather than direct disruption of protein sequence. Third, disease‐relevant transcripts may not correspond to canonical reference transcripts. Finally, integration of transcriptomic and functional evidence is often required to establish biological relevance. Together, these observations demonstrate that transcript biology is not a niche consideration applicable to a small number of exceptional genes. Rather, transcript‐dependent mechanisms represent a recurring theme across rare disease genomics and provide a compelling rationale for systematic approaches to transcript‐aware variant interpretation.

## 7. Emerging Technologies and Future Directions

The rapid expansion of transcriptomic technologies is transforming the role of transcript biology in rare disease genomics. Whereas transcript‐aware interpretation has historically relied on curated annotations and bulk expression datasets, emerging approaches now provide increasingly detailed views of transcript structure, regulation and function. These advances are narrowing the gap between variant discovery and functional characterisation and are likely to accelerate the clinical adoption of transcript‐informed variant interpretation.

### 7.1. Long‐Read Transcriptomics and Isoform Resolution

Short‐read RNA sequencing has substantially improved our understanding of alternative splicing but remains limited in its ability to reconstruct full‐length transcripts. Long‐read sequencing technologies now permit direct characterisation of complete transcript isoforms, revealing transcript structures that are difficult or impossible to resolve using conventional approaches [12, 14]. Applications in rare disease diagnostics have already demonstrated clinical utility: long‐read transcriptomic analyses have identified previously unrecognised pathogenic isoforms, clarified complex splicing events and improved interpretation of variants affecting genes with extensive transcript diversity. In disorders involving genes such as *TTN*, *ABCA4*, *USH2A* and *DMD*, long‐read sequencing has revealed transcript complexity substantially exceeding current reference annotations. As sequencing costs continue to decline and analytical pipelines mature, long‐read transcriptomics is likely to become an increasingly important component of functional variant assessment.

### 7.2. Single‐Cell and Cell Type–Specific Transcriptomics

Bulk tissue transcriptomic datasets provide valuable information regarding tissue‐level expression but cannot fully resolve cellular heterogeneity. Single‐cell transcriptomics has demonstrated that transcript usage frequently varies between distinct cellular populations within the same tissue, providing insights that are particularly relevant for neurological, retinal, immunological and developmental disorders [15]. This capability is especially important because disease‐relevant transcripts may be restricted to specific cell populations that constitute only a small proportion of a tissue; variants affecting such transcripts may appear insignificant in bulk datasets despite having major biological consequences within the relevant cellular context. The ongoing development of the Human Cell Atlas and related initiatives promises increasingly comprehensive maps of transcript expression across human tissues and developmental stages, creating new opportunities for transcript‐aware interpretation in rare disease diagnostics.

### 7.3. Functional Transcriptomics and RNA‐Based Diagnostics

RNA sequencing is increasingly transitioning from a research tool to a clinical diagnostic modality [40]. Beyond identifying aberrant splicing, transcriptomic analyses can detect exon skipping, poison exon activation, intron retention, allele‐specific expression and transcript degradation that may not be apparent from genomic sequencing alone [12]. Within ACMG/AMP interpretation, such results should be treated according to the molecular consequence they establish and the applicable ClinGen splicing framework, rather than described generically as PS3 functional evidence.

### 7.4. Artificial Intelligence and Transcript Prioritisation

The growing complexity of transcriptomic datasets creates opportunities for computational approaches capable of integrating diverse evidence streams. Machine‐learning models are increasingly being applied to splicing prediction, transcript prioritisation, protein consequence assessment and genotype–phenotype correlation [17, 24]. Future systems may combine transcript expression data, developmental transcriptomics, exon utilisation metrics, phenotype ontologies, functional datasets and genomic variation into unified predictive frameworks. Such approaches could assist in identifying disease‐relevant transcripts and prioritising variants for downstream investigation. Nevertheless, automated transcript prioritisation should complement rather than replace expert interpretation. Biological context, disease mechanism and clinical phenotype remain essential components of variant assessment that cannot currently be inferred from transcriptomic data alone.

### 7.5. Validating Transcript‐Aware Interpretation

As transcriptomics becomes increasingly integrated into clinical genomics, systematic validation of transcript‐aware interpretation frameworks will be essential. Future studies should evaluate whether incorporation of transcriptomic evidence improves diagnostic accuracy, reduces variant misclassification and enhances interlaboratory consistency, building on established approaches for measuring concordance of ACMG/AMP classification between laboratories [23]. The primary claim made for TRACE should be stated precisely, because it determines what validation would look like. The framework is intended to make transcript determinations explicit, reproducible and auditable. Improvement in classification accuracy is a hypothesis that follows from this rather than a claim made here. Prospective evaluation across transcript‐sensitive genes such as *TTN, SCN1A, SCN2A, DMD, ABCA4* and *CDKL5* may provide an effective framework for assessing clinical utility. Such studies will be critical for establishing evidence‐based standards for integrating transcript biology into routine genomic practice. A retrospective design would proceed as follows. A set of variants in transcript‐sensitive genes already classified by expert panels would be re‐curated by curators blinded to the panel classification, each variant assessed twice, once under standard practice and once under TRACE, with the order randomised across curators. The primary endpoint would be concordance with the expert panel classification. Secondary endpoints would be inter‐curator agreement measured as Cohen′s kappa, the proportion of variants of uncertain significance resolved in either direction, the proportion of transcript determinations supported by a documented rationale, and time per variant, since a framework that improves concordance at unacceptable cost will not be adopted. A prospective component would audit consecutive transcript‐triggered cases in a diagnostic service against the triage rules of Section 4.7. The result that would count as failure should also be stated: If intercurator agreement on transcript determinations does not improve under the framework, its central claim is unsupported, since reproducibility rather than accuracy is what it primarily asserts.

## 8. Conclusions

Transcript context is a genuine determinant of molecular consequence, but transcript‐aware reasoning is already embedded in several established ACMG/AMP refinements. The contribution claimed for TRACE is therefore procedural rather than evidentiary.

TRACE provides a gated workflow for recognising when transcript context is materially uncertain, escalating assessment proportionately, documenting the evidence and discordance and deciding when interpretation should stop. It does not create new evidence codes, assign transcript‐specific points or independently strengthen or weaken established criteria.

Applicable ClinGen SVI and gene/disease‐specific VCEP specifications remain authoritative for criterion application and strength. TRACE addresses the residual cross‐criterion problem: making the antecedent transcript‐context determination explicit when existing guidance does not already resolve it.

The immediate test of TRACE is reproducibility and feasibility, not an assumed increase in diagnostic yield. Retrospective and prospective validation should determine whether structured transcript‐context assessment improves agreement, reduces avoidable transcript‐related errors and can be implemented within realistic diagnostic workflows.

## Funding

No funding was received for this manuscript. Open access publishing facilitated by The University of Newcastle, as part of the Wiley ‐ The University of Newcastle agreement via the Council of Australasian University Librarians.

## Conflicts of Interest

The author declares no conflicts of interest.

## Data Availability

Data sharing is not applicable to this article as no datasets were generated or analysed during the current study.
